# Transcriptome analysis highlights the role of ferroptosis in palmitic acid–induced endothelial dysfunction

**DOI:** 10.1093/sexmed/qfac008

**Published:** 2023-01-24

**Authors:** Xiao-Hui Tan, Yang-Yang Gu, Wen-Peng Song, Tie-Gui Nan, Wei-Dong Song, Dong Fang, Yi-Ming Yuan, Zhong-Cheng Xin, Xue-Song Li, Rui-Li Guan

**Affiliations:** Department of Urology, Peking University First Hospital, Beijing 100034, PR China; Institute of Urology, Peking University, Beijing 100034, PR China; Beijing Key Laboratory of Urogenital Diseases (male) Molecular Diagnosis and Treatment Center, Beijing 100034, PR China; Institute of Urology, Peking University, Beijing 100034, PR China; Beijing Key Laboratory of Urogenital Diseases (male) Molecular Diagnosis and Treatment Center, Beijing 100034, PR China; Department of Radiation Medicine, Institute of Systems Biomedicine, School of Basic Medical Sciences, Peking University Health Science Center, Beijing 100191, PR China; Institute of Urology, Peking University, Beijing 100034, PR China; Beijing Key Laboratory of Urogenital Diseases (male) Molecular Diagnosis and Treatment Center, Beijing 100034, PR China; Department of Stomatology, Beijing Tiantan Hospital, Capital Medical University, Beijing 100070, PR China; State Key Laboratory Breeding Base of Dao-di Herbs, National Resource Center for Chinese Materia Medica, China Academy of Chinese Medical Sciences, Beijing 100700, PR China; Department of Urology, Peking University First Hospital, Beijing 100034, PR China; Institute of Urology, Peking University, Beijing 100034, PR China; Beijing Key Laboratory of Urogenital Diseases (male) Molecular Diagnosis and Treatment Center, Beijing 100034, PR China; Department of Urology, Peking University First Hospital, Beijing 100034, PR China; Institute of Urology, Peking University, Beijing 100034, PR China; Beijing Key Laboratory of Urogenital Diseases (male) Molecular Diagnosis and Treatment Center, Beijing 100034, PR China; Department of Urology, Peking University First Hospital, Beijing 100034, PR China; Institute of Urology, Peking University, Beijing 100034, PR China; Beijing Key Laboratory of Urogenital Diseases (male) Molecular Diagnosis and Treatment Center, Beijing 100034, PR China; Male Reproductive and Sexual Medicine, Department of Urology, the Second Hospital of Tianjin Medical University, Tianjin 300211, PR China; Institute of Urology, Tianjin Medical University, Tianjin 300211, PR China; Department of Urology, Peking University First Hospital, Beijing 100034, PR China; Institute of Urology, Peking University, Beijing 100034, PR China; Beijing Key Laboratory of Urogenital Diseases (male) Molecular Diagnosis and Treatment Center, Beijing 100034, PR China; Department of Urology, Peking University First Hospital, Beijing 100034, PR China; Institute of Urology, Peking University, Beijing 100034, PR China; Beijing Key Laboratory of Urogenital Diseases (male) Molecular Diagnosis and Treatment Center, Beijing 100034, PR China

**Keywords:** endothelial cells, ferroptosis, lipid metabolism, palmitic acid, RNA-seq

## Abstract

**Background:**

Palmitic acid (PA) has a lipotoxic effect on blood vessels, leading to endothelial dysfunction and cell death. The underlying mechanisms are not yet fully understood.

**Aim:**

We sought to investigate the effects of PA on endothelial cells, with an emphasis on ferroptosis.

**Methods:**

Rat corpus cavernosum endothelial cells (RCCECs) and human umbilical vein endothelial cells (HUVECs) were treated with PA to induce a pattern of cell death, as evidenced by the evaluation of cell viability. The differentially expressed genes were measured via RNA sequencing to reveal potential mechanisms. The intracellular levels of glutathione (GSH), malondialdehyde (MDA), ferrous ion (Fe^2+^), and reactive oxygen species (ROS) were evaluated using commercial kits. Western blot was performed to determine the expressions of relative proteins.

**Outcomes:**

At the end of the study period, the evaluated outcomes were cell viability, transcriptome profiles, the expressions of glutathione peroxidase 4 (GPX4) and solute carrier family 7 member 11 (SLC7A11), as well as levels of GSH, MDA, Fe^2+^, and ROS.

**Results:**

PA-induced cell death of RCCECs and HUVECs was demonstrated in a dose- and time-dependent manner. Based on the findings of RNA-sequencing (RNA-seq), enrichment of many biological processes associated with cell cycle and response to stimulus occurred. More importantly, ferroptosis was highlighted in the bioinformatic analysis of both endothelial cells. The levels of intracellular Fe^2+^, MDA, and ROS were significantly increased following PA exposure while GSH was decreased, suggesting excessive iron accumulation, development of lipid peroxidation, and imbalanced redox homeostasis. Mechanistically, PA decreased the protein expression levels of GPX4 and SLC7A11 in endothelial cells, both of which played crucial roles in ferroptotic cell death.

**Clinical Translation:**

This study suggests that ferroptosis may be a useful target for novel therapeutic interventions for endothelial dysfunction and cell death in vascular diseases such as erectile dysfunction.

**Strengths and Limitations:**

In this study, we found that ferroptosis could participate in PA-induced endothelial dysfunction and cell death. A limitation of the study is that it did not shed light on the overall mechanisms of this process. Therefore, further research on the intricate networks of regulating ferroptosis is needed.

**Conclusion:**

Overall, the occurrence of ferroptosis was demonstrated in the PA-treated HUVECs and RCCECs in this study.

## Introduction

Vascular endothelial cells (ECs), also called the vascular endothelium, constitute the interior surface of arteries, veins, and capillaries, which are in direct contact with diverse components and cells of blood.^1^ ECs perform many critical functions in controlling tissue homeostasis, including angiogenesis, regulation of vascular tone, blood coagulation, and trafficking of immune cells.^2^ More importantly, the impairment of ECs has played an essential role in the pathogenesis of several cardiovascular and endocrinological diseases. For instance, endothelial dysfunction could be an early feature of stroke, diabetes, and atherosclerosis.^3^^,^ ^4^ Increased plasma free fatty acids (FFAs) are associated with obesity and type 2 diabetes mellitus and may reduce nitric oxide production, triggering the onset of endothelial dysfunction.^5^^,^  ^6^ Palmitic acid (PA) is one of the most abundant FFAs in the human body and often occurs in the daily diet. PA has been reported to lead to aggravated apoptosis of vascular ECs, which has attracted increasing attention owing to its lipotoxic effect on blood vessels.^6^ Recently, rapid progress has occurred in understanding of related mechanisms, such as excessive generation of reactive oxygen species (ROS), impaired insulin signaling, and upregulation of inflammatory signaling. Therefore, modulation of the involved pathways may protect against endothelial dysfunction and provide a favorable cardiovascular outcome.

Ferroptosis, a novel modality of programmed cell death dependent on iron, is characterized by an overload of lipid peroxidation products and excessive iron accumulation, which is generally accompanied by increased lipid peroxide and ROS production.^7^ It has been revealed that system X_c_^−^ and glutathione peroxidase 4 (GPX4) are critically important in the suppression of ferroptosis.^8^ System X_c_^−^ is a transmembrane protein complex containing the subunit solute carrier family 7 member 11 (SLC7A11), whose blockage can lead to the depletion of glutathione (GSH). In addition, GPX4 is the major enzyme catalyzing the reduction of GSH, protecting cells against ferroptosis by converting lipid peroxides into nontoxic lipid alcohols.^9^ Recent studies have demonstrated that ferroptosis has been linked to various pathologies associated with tissue damage and subsequent cell loss, such as tumors, ischemia–reperfusion injury, and neurological diseases.^8^ However, the role of ferroptosis in the dysfunction and cell death of ECs stimulated by PA remains to be elucidated.

In this study, we aimed to investigate the potential mechanisms of PA-induced endothelial dysfunction and cell death, determining whether ferroptosis is involved in this process. Our findings have shown that PA can lead to ferroptotic cell death, providing new insight into the role of ferroptosis in related diseases.

## Materials and methods

### Cell culture and reagents

Human umbilical vein ECs (HUVECs; Cat# 8000, ScienCell, Carlsbad, CA, USA) and rat corpus cavernosum ECs (RCCECs; Cat# CP-R133, Procell, Wuhan, China) were cultured in EC medium (Cat# 1001, ScienCell) that contained fetal bovine serum (5%; Cat# 0025, ScienCell), EC growth supplement (1%; Cat# 1052, ScienCell), and antibiotic solutions (1%, penicillin/streptomycin; Cat# 0503, ScienCell). Primary cells were incubated in a humidified atmosphere with 5% CO_2_ at 37°C and served for later experiments.

PA working solution (10 mM) was prepared as described previously.^10^^,^  ^11^ Briefly, fine-powdered PA (Cat# HY-N0830, MCE, Shanghai, China) was fully dissolved in ethanol, mixed with 10% fatty acid-free bovine serum albumin (Cat# A8850, Solarbio, Beijing, China), co-incubated at 55°C in a water bath, filtered with 0.45-μm membranes, aliquoted into fractions, and stored at −80°C for up to 6 months.

### RNA isolation, library preparation, and RNA sequencing

Total RNA from PA-treated or untreated ECs was isolated using TRIzol reagent (Invitrogen, Carlsbad, CA, USA) according to the manufacturer’s instructions. The libraries were constructed with the mRNA-seq Lib Prep Kit (Cat# RK20302, ABclonal, Wuhan, Hubei, China) and sequenced on an Illumina NovaSeq 6000 platform using 150-bp paired-end sequencing mode.

The raw files were assessed for initial quality using FastQC (version 0.11.7), trimmed for adapter sequences using Cutadapt (version 2.7), and aligned to the human (GRCh38.102/hg38) and rat (Rnor_6.0.102/rn6) reference genome using Hisat2 (version 2.0.5). Transcript assembly was carried out using Stringtie. The gene abundance was expressed as the fragments per kilobase of transcript per million reads mapped. The differentially expressed genes (DEGs) were analyzed with Gene Ontology (GO) and Kyoto Encyclopedia of Genes and Genomes (KEGG) to identify enriched pathways and potential functions.

### Cell viability analysis

HUVECs and RCCECs were seeded in a 96-well plate format for 24 hours and treated with PA (up to 1.0 mM) at 37°C and 5% CO_2_. The cell viability was measured using a Cell Counting Kit-8 (CCK-8; Cat# KGA317, Keygen Biotech, Nanjing, China), with CCK-8 solution (10 μL/well) added to the cells. After a 2-hour incubation at 37°C and 5% CO_2_, absorbance at 450 nm was determined with a microplate reader (ThermoFisher Scientific, Waltham, MA, USA).

### Western blot

ECs were harvested and then treated with ice-cold RIPA lysis buffer (Cat# KGP702, Keygen Biotech) containing PMSF (Cat# KGP610, Keygen Biotech) and a protease and phosphatase inhibitor cocktail (Cat# PPC1010, Sigma-Aldrich, St. Louis, MO, USA). The proteins were separated using 10%–12% SDS-PAGE (sodium dodecyl sulfate-polyacrylamide gel electrophoresis) gels and transferred to polyvinylidene difluoride membranes (Millipore, Bedford, MA, USA). The blots were blocked with 5% skim milk for 60 minutes at room temperature, incubated with corresponding primary antibodies and secondary antibodies, and detected by enhanced chemiluminescence using the Syngene G-Box systems (Syngene, Cambridge, UK). The band intensity was quantified using ImageJ software (NIH, Bethesda, MD, USA) with β-actin as a loading control. The following primary antibodies were used in this study: GPX4 (Cat# 67763–1-Ig, Proteintech, Rosemont, IL, USA; RRID: AB_2909469), SLC7A11 (Proteintech Cat# 26864–1-AP, RRID: AB_2880661), β-ACTIN (Proteintech Cat# 66009–1-Ig, RRID: AB_2687938).

### Determination of ROS production

Intracellular ROS production was determined using an ROS assay kit (Cat# S0033; Beyotime Biotech, Shanghai, China). The ECs were exposed to the indicated conditions and then incubated with DCFH-DA (10 μM) for 30 min at 37°C and 5% CO_2_. After being washed with phosphate-buffered saline (PBS), the cells were observed under a fluorescence microscope and the intensity was quantified using ImageJ software.

### Measurement of Fe ^2+^, MDA, and GSH

According to the manufacturer’s protocols, the intracellular values of ferrous ion (Fe^2+^), malondialdehyde (MDA), and GSH in ECs were measured using FerroOrange (Cat# F374; Dojindo Laboratories, Kumamoto, Japan), a lipid peroxidation MDA assay kit (Cat# S0131S; Beyotime Biotech), and a GSH assay kit (Cat# S0052; Beyotime Biotech).

### Statistical analysis

The data are presented as mean (SD). Comparisons between 2 groups were made with the unpaired Student t-test. One-way ANOVA followed by Tukey’s multiple comparisons or Kruskal-Wallis test were used to determine an overall difference among various groups. Statistical analyses were performed using GraphPad Prism, version 9.0 (GraphPad Software, San Diego, CA, USA). *P* values less than 0.05 were considered significant.

## Results

### PA induced EC death

**Figure 1:**
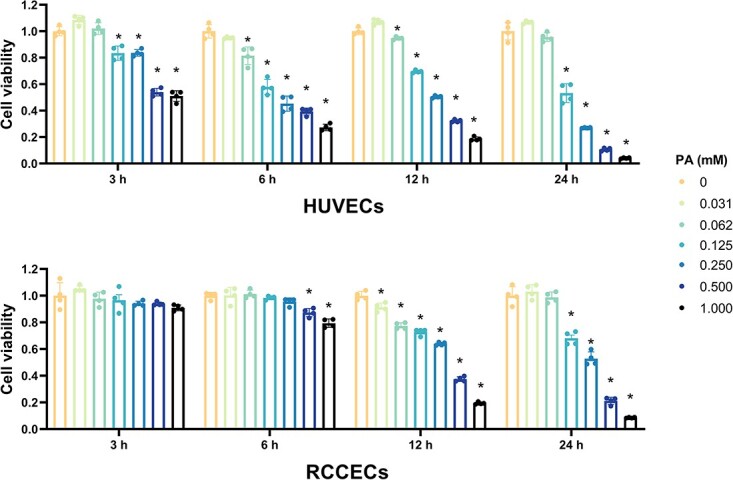
Cell death attributed to PA exposure in HUVECs and RCCECs. The cells were treated with PA for indicated conditions in a conventional incubator, and then the cell viability was measured via a CCK-8 assay (n = 4), mean (SD); ^*^P < 0.05. CCK-8, cell counting kit-8; HUVEC, human umbilical vein endothelial cell; RCCEC, rat corpus cavernosum endothelial cell.

To explore the effect of PA on the cell death of ECs, different concentrations of PA were used to treat HUVECs and RCCECs at different time points. The CCK-8 assay showed that compared with negative controls cell viability was decreased after PA exposure, depending on its concentrations and duration (Figure 1). Specifically, PA (0.25 mM, 24 hours) caused approximately 60% and 50% of cell death in HUVECs and RCCECs, respectively.

### Ferroptosis was enriched based on the RNA-seq analysis

**Figure 2:**
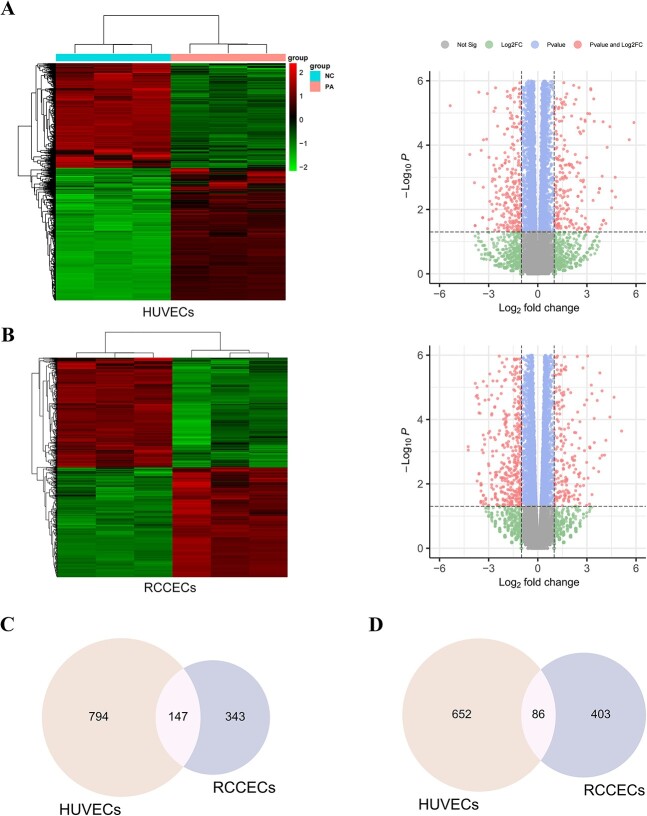
Identification of differentially expressed genes stimulated by PA in HUVECs and RCCECs. Heatmap and volcano plot of RNA-Seq data indicates the DEG patterns of PA exposure (PA, n = 3) and control group (NC, n = 3) in HUVECs (A) and RCCECs (B). The Venn diagram shows the upregulated (C) and downregulated (D) DEGs identified in 2 ECs. DEG, differentially expressed gene; EC, endothelial cell; HUVEC, human umbilical vein endothelial cell; NC, negative control; PA, palmitic acid; RCCEC, rat corpus cavernosum endothelial cell; RNA-Seq, RNA sequencing.

**Figure 3:**
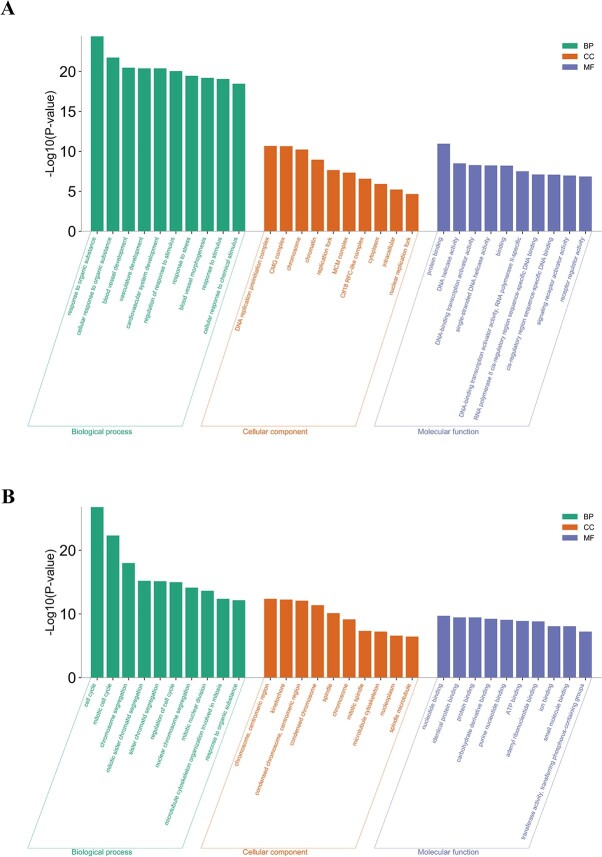
The GO analysis based on RNA sequencing. The enriched GO terms for the DEGs in HUVECs (A) and RCCECs (B). The enriched GO terms were ranked in ascending order of *P* values from left to right, indicating that the *P* value of the first column in each section was the minimum. DEG, differentially expressed gene; GO, gene ontology; HUVEC, human umbilical vein endothelial cell; RCCEC, rat corpus cavernosum endothelial cell.

To identify the molecular mechanism involved in PA-induced cell death, RNA-seq analysis was performed to determine the DEGs in 2 types of ECs between the PA-treated group and negative controls (Figure 2). A total of 1679 DEGs were identified in HUVECs and 979 in RCCECs, suggesting that HUVECs and RCCECs are sensitive to PA exposure. GO functional analysis indicated that DEGs were enriched in biological processes associated with the cell cycle and response to stimulus, including response to organic substances, regulation of cell cycle, and chromosome segregation (Figure 3). Furthermore, molecular binding was also suggested to participate in the cell damage caused by PA.

**Figure 4:**
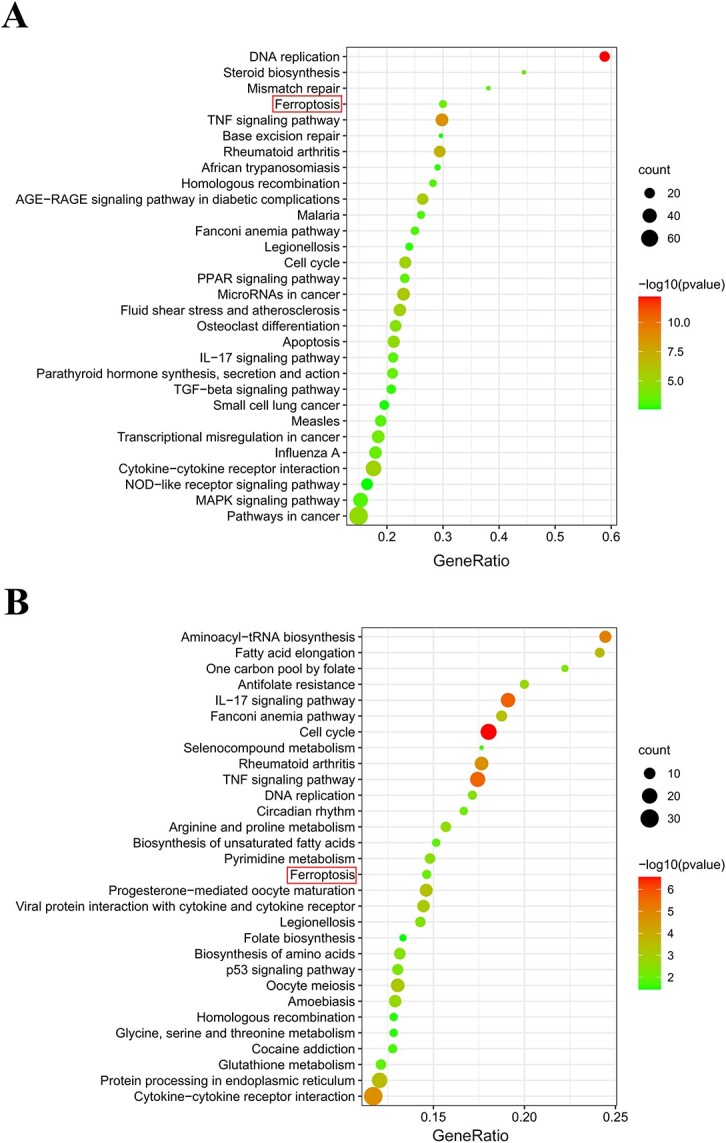
The KEGG analysis based on RNA sequencing. The top 30 KEGG pathways for the DEGs in HUVECs (A) and RCCECs (B) are illustrated using a bubble plot. DEG, differentially expressed gene; HUVEC, human umbilical vein endothelial cell; KEGG, Kyoto Encyclopedia of Genes and Genomes; RCCEC, rat corpus cavernosum endothelial cell.

The enrichment analysis of KEGG demonstrated that the DEGs significantly mapped to multiple cellular pathways of inflammation, apoptosis, and metabolism (Figure 4). Notably, ferroptosis was predicted in both EC cell types, indicating its essential function in PA-induced cell death. Meanwhile, ferroptosis-related pathways such as GSH metabolism and p53 signaling pathways were also enriched in the KEGG analysis.

### PA triggered the ferroptosis of ECs

**Figure 5:**
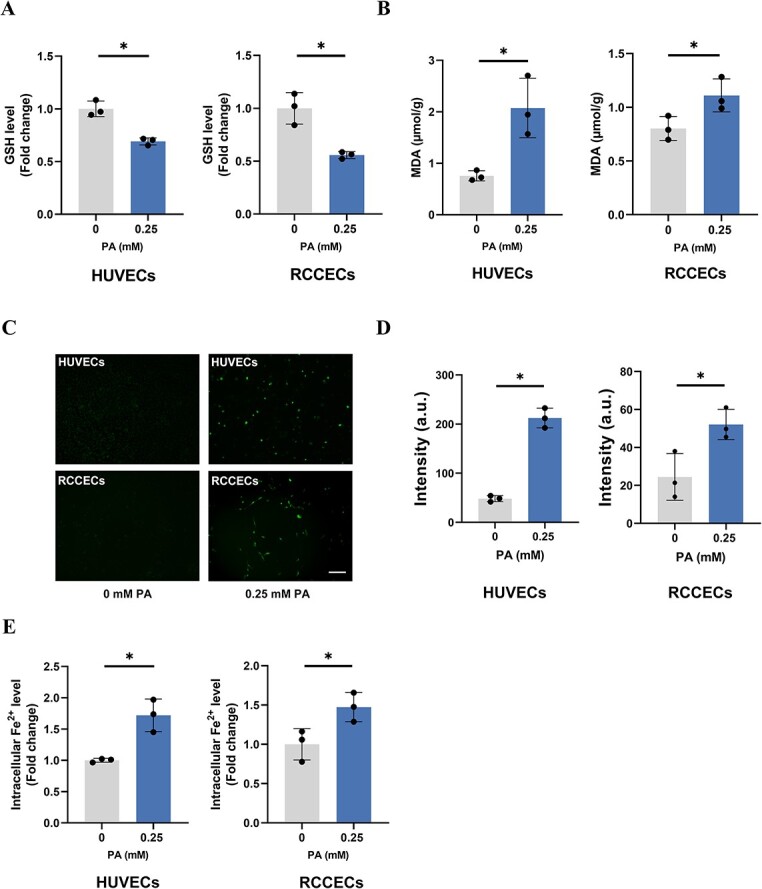
Ferroptosis induced by PA exposure in HUVECs and RCCECs. HUVECs and RCCECs were exposed to PA (0.25 mM), followed by the measurement of intracellular levels of GSH (A), MDA (B), ROS (C, D), and Fe^2+^ (E) (n = 3) mean (SD). Bar indicates 100 μm. ^*^P < 0.05.

To determine whether PA can induce the ferroptosis of ECs, we sought to detect the levels of lipid peroxidation and intracellular ROS after treatment with 0.25 mM PA. We observed that ROS production and levels of MDA were significantly increased following PA exposure whereas GSH was decreased in both ECs, suggesting the development of lipid peroxidation and imbalanced redox homeostasis (Figure 5A-D). Moreover, PA induced a notable increase of intracellular Fe^2+^ levels compared to negative controls (Figure 5E). These findings were consistent with established knowledge regarding ferroptosis under specific biological contexts.

**Figure 6:**
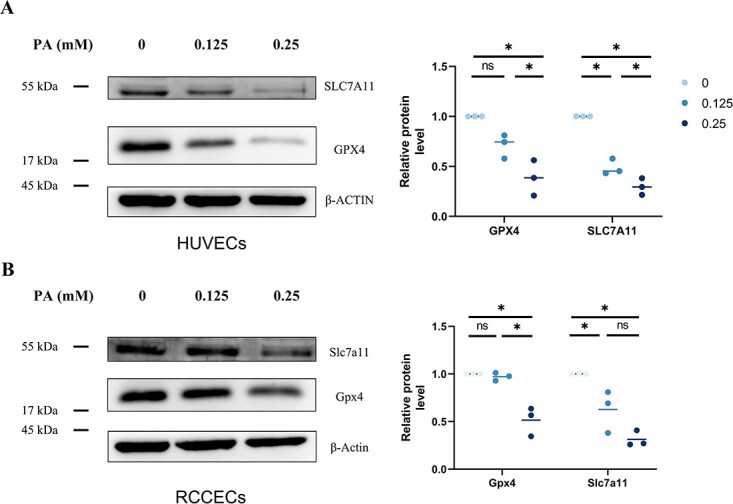
The effect of PA on ferroptosis-related proteins in endothelial cells. Protein expression levels of GPX4 and SLC7A11 in HUVECs (A) and RCCECs (B) were determined by Western blot (n = 3) mean (SD). ^*^P < 0.05; NS, *P* > 0.05. GPX4, glutathione peroxidase 4; HUVEC, human umbilical vein endothelial cell; PA, palmitic acid; RCCEC, rat corpus cavernosum endothelial cell; SLC7A11, solute carrier family 7 member 11.

Because the Cyst(e)ine/GSH/GPX4 axis represents a canonical ferroptosis-controlling pathway, we investigated the expression of related proteins. As expected, the protein expression level of GPX4 was significantly reduced in HUVECs and RCCECs treated with PA (0.25 mM, 24 hours compared with untreated cells (Figure 6). In addition, the protein expression of SLC7A11, a key component of the system X_c_^−^ cystine/glutamate antiporter responsible for cellular uptake of extracellular cystine in exchange for intracellular glutamate, was also decreased by PA treatment, even at a lower concentration. Collectively, these data suggested that PA triggered ferroptosis in HUVECs and RCCECs.

## Discussion

**Figure 7:**
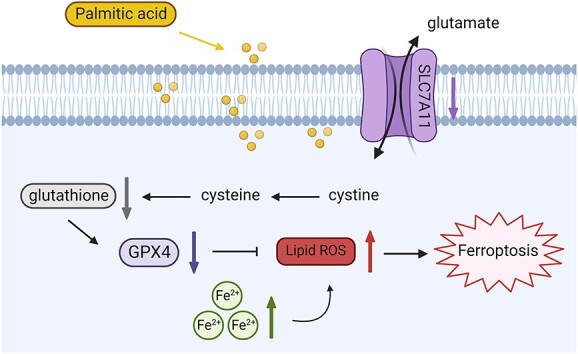
Schematic diagram of ferroptosis induced by PA in endothelial cells. PA, palmitic acid.

Endothelial cell dysfunction, injury, and death remain the leading causes of diverse pathological settings such as cardiovascular diseases, erectile dysfunction, and ischemia–reperfusion injuries.^12-14^ A growing number of published studies in this field suggest that the accumulation of lipids can pose a threat to the normal function of ECs.^15^^,^ ^16^ Proinflammatory molecules can be generated in ECs by saturated FFAs such as myristic acid (C14:0), PA (C16:0), and stearic acid (C18:0), which may be mediated through the alteration of cell membrane properties to activate Toll-like receptors.^17^ FFAs are also principal sources of ROS accumulation, which leads to oxidative stress. Moreover, saturated FFAs can promote apoptosis of ECs via nuclear factor–kappaB activation.^18^ Recent studies have focused on the role of ferroptosis, a newly discovered form of programmed cell death, in certain pathological contexts. Mounting evidence has demonstrated that ferroptosis has unique network mechanisms and functions, which are different from necrosis, apoptosis, and autophagy. Dysregulated lipid metabolism, especially unrestrained phospholipid peroxidation, has been the hallmark of ferroptosis.^8^ In the present study, we demonstrated the occurrence of ferroptosis within HUVECs and RCCECs exposed to PA, providing preliminary evidence for the role of ferroptosis in endothelial dysfunction and cell death induced by high levels of FFAs **(**Figure 7**)**.

Saturated FFAs are inevitable in Western dietary patterns, particularly in animal-based foods. For example, PA and stearic acids are predominant in butter, palm kernel oil, meat, and dairy products. Compared with unsaturated FFAs, they are thought to be associated with a significantly increased risk for cardiovascular diseases.^19^ Reducing the consumption of saturated fat has been prioritized in current public health dietary recommendations for the improvement of cardiovascular health.^20^ It has been established that elevated levels of plasma FFAs often cause a detrimental imbalance in cell metabolism.^5^ Previous studies have shown that PA-induced EC death has been involved with various signaling pathways, including autophagy, apoptosis, and necroptosis.^18^^,^ ^21^ However, the underlying mechanisms are not yet fully understood. In this study, HUVECs and RCCECs were used to identify other possible forms of cell death involved in this process. The cell viability of PA-induced ECs was significantly reduced compared with that of untreated cells, indicating that EC death was successfully elicited. The findings of RNA-seq suggest the involvement of ferroptosis in HUVECs and RCCECs following the stimulation of PA. Wang et al. reported the development of ferroptosis in cardiomyocytes exposed to PA, in which HSF1 may serve as a key defender against ferroptotic cell death.^22^ Moreover, PA-induced ferroptosis was also observed in numerous types of cells, including vascular smooth muscle cells and hepatocytes.^23^^,^  ^24^ Therefore, PA-mediated ferroptosis in endothelial dysfunction and cell death was taken into consideration in the present study.

Extensive investigations of ferroptosis over the past 20 years have rapidly broadened our understanding of this form of cell death, which is mainly executed by phospholipid peroxidation resulting from impaired metabolism of iron, ROS, and phospholipids. Initially, mechanisms governing ferroptosis are centered around cysteine and GSH metabolism as well as the activity and stability of GPX4.^25^ In addition, the system X_c_^−^ is an amino acid antitransporter composed of 2 subunits, SLC7A11 and SLC3A2, which affect the action of glutathione peroxidases and the synthesis of GSH.^26^ The downregulation of system X_c_^−^ and GPX4 has been implicated in the ferroptotic response of cancer cells.^25^^,^  ^27^ In this study, expression of GPX4 and SLC7A11 was reduced in the ECs treated with PA, suggesting the promotion of ferroptosis in this setting. After the stimulation of PA, HUVECs and RCCECs produced excessive ROS and MDA and significantly inhibited the biosynthesis of GSH, which reflected the sustained activation of lipid peroxidation and oxidative stress. It has been reported that zinc oxide nanoparticles can induce ferroptosis of HUVECs, as evidenced by the assessment of ROS production, lipid peroxidation, and cell viability.^28^ Additionally, ferroptosis depends on macroautophagy/autophagy and is mediated by ferritinophagy in such a context. As the biological functions of ferroptosis have been depicted to some extent, its pharmacological modulation, via either inhibition or induction, may yield promising clinical outcomes for certain diseases. For example, it has been demonstrated that metformin and GSK-J4 may ameliorate lipotoxicity to cardiomyocytes via ferroptosis.^23^^,^  ^29^ Quercetin, which decreases the expression of ATF3, could alleviate acute kidney injury via inhibition of ferroptosis.^30^ Overall, ferroptosis might play an important role in PA-treated endothelial dysfunction and cell death.

Several principal limitations should be acknowledged in our study. First, endothelial dysfunction and cell death owing to acute PA infusion differ from the chronic process in the human body, although elevated levels of plasma FFAs are a remarkable risk factor for vascular diseases. Second, the lack of other types of ECs and animal model studies may not illustrate the entire mechanisms of ferroptosis under these pathological conditions. Third, ferroptosis can be regulated via intricate networks that are more diverse than originally supposed. Recent advances have uncovered GPX4-independent mechanisms of ferroptosis surveillance, including NADPH/FSP1/CoQ10 and GCH1/BH4 pathways.^8^ More evidence is required to address these issues for potential discoveries in this exciting field.

## Conclusions

In summary, ferroptosis occurrence was implicated in the PA-treated HUVECs and RCCECs based on the findings of RNA-seq and related biochemical investigations. Further research should be directed at ferroptosis in the development of endothelial cytotoxicity and dysfunction, which might be a potential target for novel therapeutic interventions.

## Author contributions

Conception and design: R.-L.G. Administrative support: Z.-C.X., X.-S.L.; Collection and assembly of data: X.-H.T., Y.-Y.G., W.-P.S., T.-G.N., W.-D.S., D.F., Y.-M.Y. Data analysis and interpretation: X.-H.T., Y.-Y.G., W.-P.S. Writing–original draft: X.-H.T. Writing–review and editing: R.-L.G. Final approval of manuscript: all authors.

## Funding

The work was supported by the National Natural Science Foundation of China (grant no. 81971379).

*Conflicts of interest:* The authors declare no potential conflicts of interest.

## Data availability

All data generated or analyzed during this study are included in this published article and its supplementary information files, which are available on reasonable request. RNA-Seq data have been made available in the *Gene Expression Omnibus* (GEO) database (http://www.ncbi.nlm.nih.gov/geo/) under the accession number *GSE205913*.

## References

[1] McCarron JG, Lee MD, Wilson C. The endothelium solves problems that endothelial cells do not know exist. Trends Pharmacol Sci. 2017;38:322–338 28214012 10.1016/j.tips.2017.01.008 PMC5381697

[2] Monteiro JP, Bennett M, Rodor J, Caudrillier A, Ulitsky I, Baker AH. Endothelial function and dysfunction in the cardiovascular system: the long non-coding road. Cardiovasc Res. 2019;115:1692–1704 31214683 10.1093/cvr/cvz154 PMC6755355

[3] Sashindranath M, Nandurkar HH. Endothelial dysfunction in the brain. Stroke. 2021;52:1895–1904 33794655 10.1161/STROKEAHA.120.032711 PMC8078121

[4] Kaur R, Kaur M, Singh J. Endothelial dysfunction and platelet hyperactivity in type 2 diabetes mellitus: molecular insights and therapeutic strategies. Cardiovasc Diabetol. 2018;17:121 30170601 10.1186/s12933-018-0763-3 PMC6117983

[5] Mallick R, Duttaroy AK. Modulation of endothelium function by fatty acids. Mol Cell Biochem. 2021;477:15–38 34529222 10.1007/s11010-021-04260-9 PMC8755678

[6] Ghosh A, Gao L, Thakur A, Siu PM, Lai CWK. Role of free fatty acids in endothelial dysfunction. J Biomed Sci. 2017;24:50 28750629 10.1186/s12929-017-0357-5 PMC5530532

[7] Yang WS, Stockwell BR. Ferroptosis: death by lipid peroxidation. Trends Cell Biol. 2016;26:165–176 26653790 10.1016/j.tcb.2015.10.014 PMC4764384

[8] Jiang X, Stockwell BR, Conrad M. Ferroptosis: mechanisms, biology and role in disease. Nat Rev Mol Cell Biol. 2021;22:266–282 33495651 10.1038/s41580-020-00324-8 PMC8142022

[9] Stockwell BR, Jiang X, Gu W. Emerging mechanisms and disease relevance of ferroptosis. Trends Cell Biol. 2020;30:478–490 32413317 10.1016/j.tcb.2020.02.009 PMC7230071

[10] Fratantonio D, Speciale A, Ferrari D, Cristani M, Saija A, Cimino F. Palmitate-induced endothelial dysfunction is attenuated by cyanidin-3-O-glucoside through modulation of Nrf2/Bach1 and NF-κB pathways. Toxicol Lett. 2015;239:152–160 26422990 10.1016/j.toxlet.2015.09.020

[11] Gu Y, Tan X, Song W et al. Icariside II attenuates palmitic acid-induced endothelial dysfunction through SRPK1-Akt-eNOS Signaling Pathway. Front Pharmacol. 2022;13:920601 35846993 10.3389/fphar.2022.920601 PMC9280058

[12] Pincus J, Sandoval V, Dick B et al. E-cigarette-associated endothelial damage: a potential mechanism for erectile dysfunction. Sex Med Rev. 2022;10:168–173 33931382 10.1016/j.sxmr.2021.01.003

[13] Paone S, Baxter AA, Hulett MD, Poon IKH. Endothelial cell apoptosis and the role of endothelial cell-derived extracellular vesicles in the progression of atherosclerosis. Cell Mol Life Sci. 2019;76:1093–1106 30569278 10.1007/s00018-018-2983-9 PMC11105274

[14] Méndez-Carmona N, Wyss RK, Arnold M et al. Differential effects of ischemia/reperfusion on endothelial function and contractility in donation after circulatory death. J Heart Lung Transpl. 2019;38:767–777 10.1016/j.healun.2019.03.004 30952549

[15] Bai T, Li M, Liu Y, Qiao Z, Wang Z. Inhibition of ferroptosis alleviates atherosclerosis through attenuating lipid peroxidation and endothelial dysfunction in mouse aortic endothelial cell. Free Radical Bio Med. 2020;160:92–102 32768568 10.1016/j.freeradbiomed.2020.07.026

[16] Li M, van Esch B, Wagenaar G, Garssen J, Folkerts G, Henricks PAJ. Pro- and anti-inflammatory effects of short chain fatty acids on immune and endothelial cells. Eur J Pharmacol. 2018;831:52–59 29750914 10.1016/j.ejphar.2018.05.003

[17] Goldberg IJ, Bornfeldt KE. Lipids and the endothelium: bidirectional interactions. Curr Atheroscler Rep. 2013;15:365 24037142 10.1007/s11883-013-0365-1 PMC3825167

[18] Staiger K, Staiger H, Weigert C, Haas C, Häring HU, Kellerer M. Saturated, but not unsaturated, fatty acids induce apoptosis of human coronary artery endothelial cells via nuclear factor-κB activation. Diabetes. 2006;55:3121–3126 17065351 10.2337/db06-0188

[19] Sacks FM, Lichtenstein AH, Wu JHY et al. Dietary Fats and Cardiovascular Disease: A Presidential Advisory From the American Heart Association. Circulation. 2017;136:e1–e23 28620111 10.1161/CIR.0000000000000510

[20] Lichtenstein AH, Appel LJ, Vadiveloo M et al. 2021 Dietary Guidance to Improve Cardiovascular Health: a scientific statement from the American Heart Association. Circulation. 2021;144:e472–e487 34724806 10.1161/CIR.0000000000001031

[21] Khan MJ, Rizwan Alam M, Waldeck-Weiermair M et al. Inhibition of autophagy rescues palmitic acid-induced necroptosis of endothelial cells. J Biol Chem. 2012;287:21110–21120 22556413 10.1074/jbc.M111.319129 PMC3375534

[22] Wang N, Ma H, Li J et al. HSF1 functions as a key defender against palmitic acid-induced ferroptosis in cardiomyocytes. J Mol Cell Cardiol. 2021;150:65–76 33098823 10.1016/j.yjmcc.2020.10.010

[23] Ma W, Sun X, Zhu Y, Liu NF. Metformin attenuates hyperlipidaemia-associated vascular calcification through anti-ferroptotic effects. Journal of free radicals in biology & medicine. 2021;165:229–242 10.1016/j.freeradbiomed.2021.01.033 33513420

[24] Qi J, Kim J, Zhou Z, Lim CW, Kim B. Ferroptosis affects the progression of nonalcoholic steatohepatitis via the modulation of lipid peroxidation–mediated cell death in mice. Am J Pathol. 2020;190:68–81 31610178 10.1016/j.ajpath.2019.09.011

[25] Yang WS, SriRamaratnam R, Welsch ME et al. Regulation of Ferroptotic cancer cell death by GPX4. Cell. 2014;156:317–331 24439385 10.1016/j.cell.2013.12.010 PMC4076414

[26] Liu J, Xia X, Huang P. xCT: a critical molecule that links cancer metabolism to redox signaling. Mol Ther. 2020;28:2358–2366 32931751 10.1016/j.ymthe.2020.08.021 PMC7647670

[27] Jiang L, Kon N, Li T et al. Ferroptosis as a p53-mediated activity during tumour suppression. Nature. 2015;520:57–62 25799988 10.1038/nature14344 PMC4455927

[28] Qin X, Zhang J, Wang B et al. Ferritinophagy is involved in the zinc oxide nanoparticles-induced ferroptosis of vascular endothelial cells. Autophagy. 2021;17:4266–4285 33843441 10.1080/15548627.2021.1911016 PMC8726675

[29] Xu K, Liu X, Wen B et al. GSK-J4, a specific histone lysine demethylase 6A inhibitor, ameliorates lipotoxicity to cardiomyocytes via preserving H3K27 methylation and reducing ferroptosis. Front Cardiovasc Med. 2022;9:907747 35722096 10.3389/fcvm.2022.907747 PMC9200982

[30] Wang Y, Quan F, Cao Q et al. Quercetin alleviates acute kidney injury by inhibiting ferroptosis. J Adv Res. 2021;28:231–243 33364059 10.1016/j.jare.2020.07.007 PMC7753233

